# A Functional Alternative Splicing Mutation in *AIRE* Gene Causes Autoimmune Polyendocrine Syndrome Type 1

**DOI:** 10.1371/journal.pone.0053981

**Published:** 2013-01-08

**Authors:** Junyu Zhang, Hongbin Liu, Zhiyuan Liu, Yong Liao, Luo Guo, Honglian Wang, Lin He, Xiaodong Zhang, Qinghe Xing

**Affiliations:** 1 Children’s Hospital and Institutes of Biomedical Sciences, Fudan University, Shanghai, China; 2 Bio-X Institutes, Key Laboratory for the Genetics of Developmental and Neuropsychiatric Disorders (Ministry of Education), Shanghai Jiao Tong University, Shanghai, China; 3 Henan Provincial Corps Hospital, Chinese’s Armed Police Forces, Zhengzhou, China; 4 Chongqing Municipal Corps Hospital, Chinese’s Armed Police Forces, Chongqing, China; 5 Neuroscience & Behavioral Disorders Program, Duke-NUS Graduate Medical School Singapore, Singapore; 6 Department of Physiology, National University of Singapore, Singapore; 7 Department of Psychiatry and Behavioral Sciences, Duke University Medical Center, United States of America; University of Bristol, United Kingdom

## Abstract

Autoimmune polyendocrine syndrome type 1 (APS-1) is a rare autosomal recessive disease defined by the presence of two of the three conditions: mucocutaneous candidiasis, hypoparathyroidism, and Addison’s disease. Loss-of-function mutations of the autoimmune regulator (*AIRE*) gene have been linked to APS-1. Here we report mutational analysis and functional characterization of an *AIRE* mutation in a consanguineous Chinese family with APS-1. All exons of the *AIRE* gene and adjacent exon-intron sequences were amplified by PCR and subsequently sequenced. We identified a homozygous missense *AIRE* mutation c.463G>A (p.Gly155Ser) in two siblings with different clinical features of APS-1. *In silico* splice-site prediction and minigene analysis were carried out to study the potential pathological consequence. Minigene splicing analysis and subsequent cDNA sequencing revealed that the *AIRE* mutation potentially compromised the recognition of the splice donor of intron 3, causing alternative pre-mRNA splicing by intron 3 retention. Furthermore, the aberrant *AIRE* transcript was identified in a heterozygous carrier of the c.463G>A mutation. The aberrant intron 3-retaining transcript generated a truncated protein (p.G155fsX203) containing the first 154 AIRE amino acids and followed by 48 aberrant amino acids. Therefore, our study represents the first functional characterization of the alternatively spliced *AIRE* mutation that may explain the pathogenetic role in APS-1.

## Introduction

Autoimmune polyendocrine syndrome type 1 (APS-1, OMIM 240300), formerly known as Autoimmune polyendocrinopathy-candidiasis-ectodermal dystrophy (APECED), is a rare but devastating primary immunodeficiency disorder, which usually manifests during childhood and adolescence [1], [2]. Clinical diagnosis for APS-1 typically requires the presence of at least two of the three hallmark conditions: chronic mucocutaneous candidiasis, hypoparathyroidism and Addison’s disease [2]. Mutations in autoimmune regulator (*AIR*E) gene have been linked to APS-1 [3], [4]. The *AIRE* gene encodes a 57 kDa transcription regulator of 545 amino acids involved in regulating autoimmunity by promoting the ectopic expression and presentation of tissue-restricted antigens during T-cell development in the thymus [5]. AIRE protein contains several distinct domains, such as a potential bipartite nuclear localization signals (NLS) consisting of amino acids 110–114 and 131–133, four interspersed LXXLL motifs, two plant homeodomain (PHD) fingers, caspase-recruitment domain (CARD), and SAND (named after Sp100, AIRE-1, NucP41/75, DEAF-1) domain [3], [4], [6], [7]. Consistent with these features, the AIRE protein is localized predominantly in the nucleus, where it potentially modulates the transcription of a variety of genes by interacting with specific DNA sequences and/or acting through epigenetic mechanisms [5], [6], [8].

Patients with APS-1 also routinely exhibit additional autoimmune diseases, including type 1 diabetes mellitus, hypothyroidism, vitiligo, alopecia, autoimmune hepatitis, pernicious anemia, and asplenism [9]. The range of these secondary disorders is broad and variable, affected siblings carrying the same mutations can develop divergent spectrums of autoimmune disorders [10]. To date, more than 70 different mutations of the *AIRE* gene have been identified in APS-1 patients, two major mutations (R257X and L323SfsX51) of which are responsible for 95% of the mutant alleles in APS-1 patients [11], [12], [13]. Although *AIRE* mutations are mostly autosomal recessive, an autosomal dominant mutation with distinct autoimmune phenotype has recently been identified [14]. However, it is noteworthy that few *AIRE* mutations have been functionally characterized [15]. Therefore, elucidating the functions of *AIRE* mutations will help to understand the pathophysiology of APS-1.

Here we report the identification of a missense mutation of *AIRE* in two siblings of a Chinese family. Moreover, functional characterization of the mutation suggested that this mutation resulted in alternative splicing of *AIRE* and a truncated protein with premature termination codon (PTC) downstream of exon 3 of *AIRE*.

## Results

### Clinical Characteristics of APS-1 Patients in a Chinese Family

**Figure 1:**
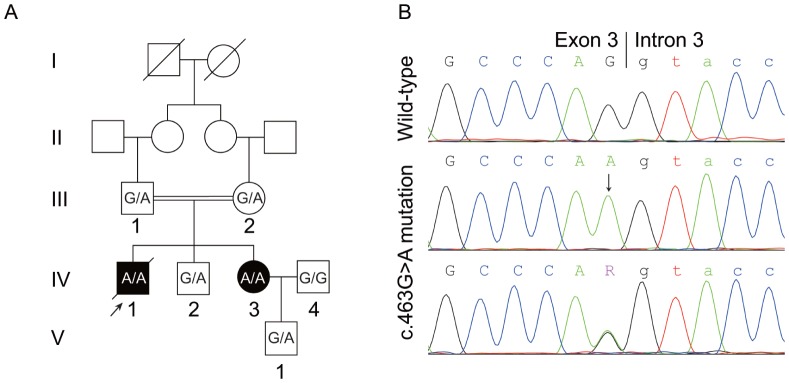
Pedigree of the two patients with APS-1 and detection of the c.463G>A *AIRE* mutation. (A) Pedigree of the Chinese family with APS-1. Numbered family members were subjected to mutational analysis. Genotypes were shown as wild-type (G/G), heterozygous (G/A) and homozygous (A/A) of the c.463G>A *AIRE* mutation. Arrow indicates the proband. (B) Direct sequence analysis of the *AIRE* gene identified homozygous carriers of c.463G>A mutation in patients, while heterozygous carriers were found in unaffected members (III-1, III-2, IV-2, V-1) of the family. Control and IV-4 carried wild-type *AIRE*. Arrow indicates the homozygous G>A mutation at the end of exon 3.

Two siblings with autosomal recessive APS-1 of a Chinese family have been examined in the study. They were born from healthy consanguineous parents of third cousins (Figure 1A). The pedigree comprised 13 individuals spanning five generations. The proband (IV-1) was a young man who was diagnosed with severe APS-1 symptoms including hypoparathyroidism, Addison’s disease, epilepsy, pernicious anemia, and chronic/tension headaches at age 18, and further developed keratopathy at age 19. At age 24, he was diagnosed with type 1 diabetes mellitus based on urine acetone body test and an increased blood glucose level (22.5 mmol/L). He died from diabetes complicated with keto acidosis 5 months later. In contrast, his sister (IV-3) who was also diagnosed with APS-1 showed mild clinical symptoms. Early signs of mucocutaneous candidiasis that can wax and wane were first appeared when she was only 1 year-old. She suffered transient Japanese encephalitis at age 7, and followed by additional symptoms, including hypoparathyroidism and epilepsy at age 15. The two patients also suffered recurrent carpopedal spasm, a common clinical manifestation of hypoparathyroidism. In contrast to her brother’s severe symptoms, the tetany has not recurred since she was age 21. The other living members of the family are healthy. A summary of the clinical manifestations of the patients are listed in Table 1.

**Table 1:** Clinical manifestations of the patients.

Patient	Sex	YOB	Manifestations (age in years at diagnosis of component)			
			MC	HP	AD	Additional components
IV-1	M	1987	–	(18)	(18)	Epilepsy (18); Pernicious anemia (18); Chronic/tension headaches (18); Keratopathy (19); Type 1 diabetes mellitus (24)
IV-3	F	1988	(1)	(15)	–	Japanese encephalitis (7); Epilepsy (15)

YOB: year of birth; MC: mucocutaneous candidiasis; HP: hypoparathyroidism; AD: Addison’s disease; numbers in parentheses represent the age in years at the diagnosis.

### Identification of *AIRE* Mutation and *in silico* Splicing Assay

Based on the clinical features of the patients, which supported the diagnosis of APS-1, mutational screening of the *AIRE* gene was carried out. Direct sequencing of the *AIRE* gene revealed that the affected patients were homozygote for a missense mutation c.463G>A at the end of exon 3, which belongs to the conserved splice donor sequence [16] (Figure 1B). The mutation was also detected in heterozygous state in the other examined healthy family members except IV-4, but was not detected in 100 ethnically matched healthy control subjects. This mutation has been previously reported in a German girl with APS-1 and authors speculated that this mutation might cause a frameshift of AIRE by skipping exon 4 [17].

While ESEfinder (http://rulai.cshl.edu/tools/ESE) [18] and SplicePort (http://spliceport.cs.umd.edu) [19] predicted splice donor site in wild-type *AIRE*, the recognition of such splice donor site was severely attenuated in the presence of c.463G>A mutation.

### The c.463G>A Mutation Generated an Aberrant Transcript Retained Intron 3

**Figure 2:**
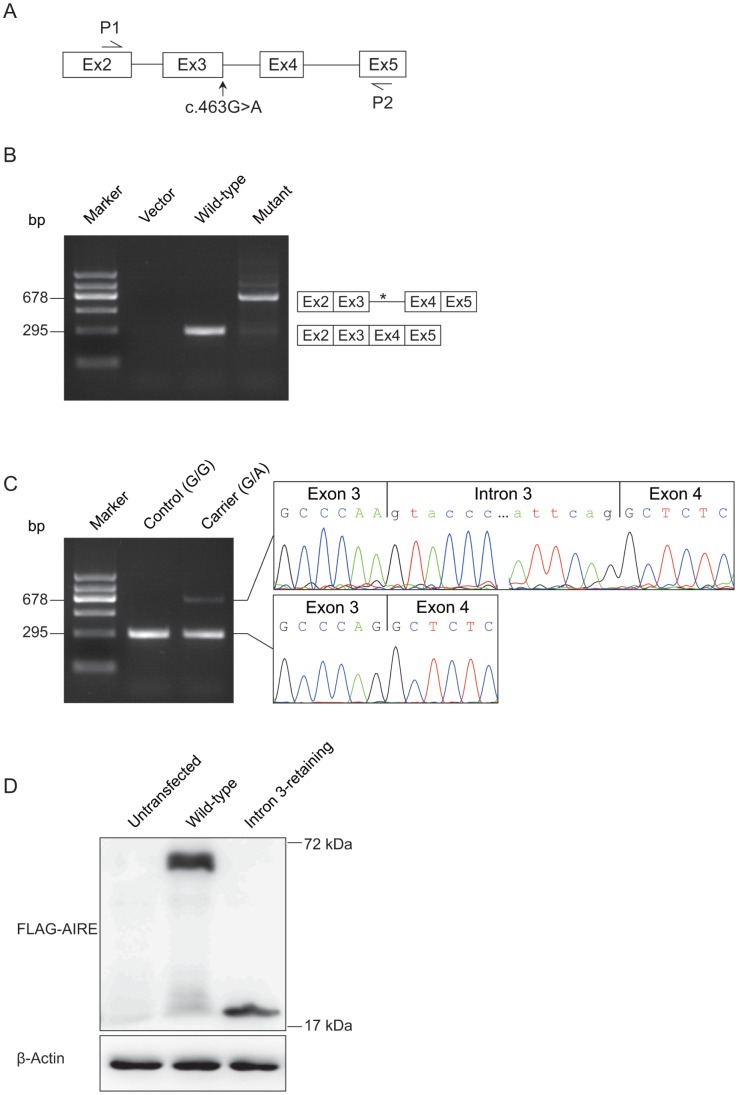
Alternative splicing of *AIRE* by the c.463G>A mutation. (A) Schematic diagram of the *AIRE* minigene constructs. Complete genomic DNA spanning from exon 2 to exon 5 of *AIRE* gene was amplified from wild-type control and the APS-1 patient (homozygous for the c.463G>A mutation), respectively, for minigene analysis. P1 and P2 were exonic primers to evaluate the results of RT-PCR. (B) Minigene constructs were individually transfected into HeLa cells. RT-PCR products amplified with the primers (P1 and P2) showed an aberrant splicing pattern in mutant minigene compared with wild-type minigene. The predicted cDNA transcripts are shown, which was confirmed by DNA sequence analysis. * indicates the premature termination codon in intron 3. (C) RT-PCR analysis of *AIRE* mRNA from lymph node needle biopsy of a heterozygous carrier (III-1; G/A) and a healthy control (G/G) using the same primers of minigene analysis. A cDNA fragment of 295-bp, which corresponded to predicted wild-type transcript, was identified in both control and the heterozygous carrier of the c.463G>A mutation. In addition, a longer 678-bp transcript was identified in the heterozygous carrier. DNA sequence analysis of the 678-bp transcript revealed the retention of exon 3. (D) *In vitro* translation assay. Total cell lysates of HEK293T cells transfected with wild-type or intron 3-retaining *AIRE* were immunoblotted with FLAG antibody. Compared with the wild-type, the intron 3-retaining *AIRE* translated a shorted product. β-actin was used as an internal control.

In order to evaluate the predicted results, we attempted to determine the exact consequence of the c.463G>A mutation in pre-mRNA splicing by *in vitro* splicing assay using a modified splicing reporter construct without the constitutively active adenovirus exonic sequences [20]. Two minigene constructs, harboring the wild-type and mutant *AIRE* fragments (1902-bp genomic DNA, spanning from *AIRE* exon 2 to exon 5) (Figure 2A), respectively, were generated. These two minigenes together with the vector were individually transfected into HeLa cells. Twenty-four hours after transfection, total RNA was extracted, reverse transcribed and amplified with primers (P1 and P2) located in exon 2 and exon 5 (Figure 2A). Differential splicing pattern was found between the cells transfected with wild-type and mutant minigenes (Figure 2B). While predicted 295-bp PCR fragment of normal splicing was shown and confirmed by sequencing analysis when wild-type minigene construct was expressed, an additional 678-bp PCR product was shown in the presence of mutant minigene construct. Sequencing analysis of the 678-bp amplicons revealed that this transcript was an alternatively spliced product which retained intron 3 (Figure 2B). Identical results were obtained using HEK293T cells (data not shown).

*AIRE* mRNA has been detected in lymph node, while whether it can be detected in peripheral blood mononuclear cells (PBMCs) has still not been reached an agreement [21]. Thus, to further confirm our results, RT-PCR analysis of total RNA extracted from lymph node needle biopsy of a heterozygous carrier (III-1; G/A) and a healthy control (IV-4; G/G) was performed with the same primers of the minigene analysis. While a 295-bp transcript was detected in both the control and heterozygous subjects, consistent with the minigene analysis result in cell lines, we specifically detected an additional aberrant 678-bp fragment in the heterozygous subject (Figure 2C). cDNA sequence analysis confirmed the 295-bp and 678-bp transcripts as wild-type and intron 3-retaining transcript, respectively (Fig. 2C). It is worthwhile to note that aberrant pre-mRNA splicing was present at much reduced level in the heterozygous carrier. Quantification of the relative proportion of wild-type and aberrant *AIRE* mRNA in lymph node of the heterozygous carrier showed that the majority (89.6%) of mRNA transcripts were wild-type suggested that the presence of degradation by the nonsense-mediated mRNA decay (NMD) [22].

Intron 3 retention was predicted to generate a truncated protein (p.G155fsX203) containing the first 154 AIRE amino acids and followed by 48 aberrant amino acids and a premature termination codon (PTC). To investigate the translation of the intron 3-retaining *AIRE* transcript, we performed immunoblotting with whole-cell lysates of transiently transfected HEK293T cells overexpressing wild-type *AIRE* mRNA, or mutated *AIRE* mRNA with the mutation c.463G>A plus a retained intron 3. When the expression construct contained the wild-type AIRE, the N-terminally tagged FLAG-AIRE protein displayed the expected molecular weight. The intron 3-retaining *AIRE* translated an abnormal protein band corresponding to the expected truncated AIRE (Figure 2D). Thus, our data demonstrated that the c.463G>A mutation reduced normal splicing by generating an alternatively spliced intron 3-retaining transcript, which resulted in a truncated protein.

## Discussion

APS-1 is an autosomal recessive disorder caused by defective of the *AIRE* gene. The disease is highly prevalent in certain genetically isolated populations, such as Finns, Sardinians and Iranian Jews [23], but is rare in Chinese population [24]. In the present study, we identified two Chinese siblings, a brother and a sister, who carried the same homozygous recurrent mutation of the *AIRE* gene, but had different onset and pattern of clinical symptoms of APS-1. Although the brother developed severe APS-1, his sister had mild symptoms. Intriguingly, the Germany girl carrying the same homozygous mutation developed severe APS-1 with three major clinical features, as well as rheumatoid factor positive arthritis, autoimmune hepatitis, chronic diarrhea, vitiligo, and hypothyroidism [17]. Therefore, it is important to emphasize that this homozygous mutation alone is not sufficient to determine all the clinical phenotypes of APS-1, suggesting that other unknown genetic and/or environmental factors in addition to AIRE dysfunction may be responsible for the diverse clinical symptoms and disease severity of APS-1.

Coding nonsynonymous mutations are commonly studied for their pathological functions through altered amino acid sequences. However, approximately 50% of all point mutations responsible for genetic diseases result in aberrant pre-mRNA splicing [16]. Of the reported *AIRE* mutations in APS-1, missense or nonsense mutations were mostly described, while splicing mutations were less common [25], [26]. Instead of a traditional approach to study AIRE (Gly155Ser) missense mutation, here we identified the potential severe pathological consequence of c.463G>A mutation, for generating an aberrant transcript retaining intron 3 (Figure 2). Because G is predominantly the last nucleotide of mammalian exons, which forms the conserved exon-intron junction [16], the c.463G>A mutation at the end of exon 3 potentially compromised the recognition of canonical GU splice donor. We hypothesized that intron 3 retention was due to severely loss-of-recognition of intron 3 splice donor, making the downstream intron 4 splice donor a favorable recognition site. It is noteworthy that the intron 3-retaining transcript was detected at much lower level than wild-type transcript in the heterozygous carrier (Figure 2C). Due to the presence of a PTC within intron 3, it is possible that NMD may play a role [22]. To support this notion, minigene constructs, which did not possess an initiation start codon, yielded predominant intron 3-retaining transcript (Figure 2A). Also, the much lower level intron 3-retaining transcript might be due to more efficient PCR amplification of the shorter fragment (Figure 2C). In addition to c.463G>A mutation, there are two more *AIRE* mutations (c.462A>T [27] and c.463+2T>C [28]) in APS-1 have been identified near this splice donor site. However, it is yet to determine whether these mutations have any effect on *AIRE* splicing. It is likely that this splice donor site might be a sensitive mutational hotspot of the *AIRE* gene. Taken together, our results revealed novel mechanism of defects in transcriptional regulation (*e.g.*, pre-mRNA splicing) of *AIRE*.

In conclusion, we report a missense *AIRE* mutation (c.463G>A) in two patients of a consanguineous Chinese family with different phenotypes of APS-1, and for the first time confirmed its pathogenetic role in altering *AIRE* pre-mRNA splicing (intron 3 retention) by minigene splicing analysis and subsequent cDNA sequencing. Our approach to study functional mutation at the genomic DNA level would widen the molecular spectrum of APS-1 and provide valuable insights into the molecular mechanisms underlying the pathophysiology of APS-1.

## Materials and Methods

### Ethics Statement

This study was approved by the Ethics Review Committee of Fudan University and conducted according to the Declaration of Helsinki Principles. Written informed consent was obtained from all participants, including the 100 healthy control individuals. The patient in this manuscript has given written informed consent (as outlined in the PLoS consent form) to publish these case details.

### Subjects

All subjects of this study were recruited from the outpatient department of Henan Provincial Corps Hospital. Peripheral blood samples were collected from 7 members of the pedigree (Figure 1A) as well as from 100 ethnically matched unrelated healthy controls. Genomic DNA was isolated from these samples according to standard techniques. Lymph node tissue samples from human inguinal region were obtained via needle biopsy of a heterozygous carrier (III-1; G/A) and a healthy control (IV-4; G/G).

### Mutational and *in silico* Splicing Analyses

All 14 exons and their flanking intron sequences of *AIRE* were analysed by direct sequencing from PCR amplicons. Primers and PCR conditions are available upon request. The mutation was identified by comparing with the reported reference sequence (NM_000383.2) and further analyzed in the 100 control individuals.

Online splice-site and cDNA sequence prediction were carried out by ESEfinder (http://rulai.cshl.edu/tools/ESE) and SplicePort (http://spliceport.cs.umd.edu). Both wild-type sequence and altered sequence were analyzed. The prediction was performed using default settings.

### Minigene Constructs and Minigene Splicing Assay

Wild-type and mutated fragments of *AIRE* (1902-bp genomic DNA, spanning from *AIRE* exon 2 to exon 5) were generated by PCR amplification from genomic DNA of normal individuals and the APS-1 patient, using the following primers: *AIRE*-*Eco*R I-F 5′-CCGGAATTCGAGACGCTTCATCTGAAGGAA-3′ and *AIRE*-*Hind* III-R 5′-CCCAAGCTTCTGACTCAAACACCTGCTGGAT-3′. The PCR fragments were digested with *Eco*R I and *Hind* III, and subsequently cloned into a modified version of the pcDNA3 minigene [20] and verified by sequencing analysis.

HeLa and HEK293T cell lines (Cell Resource Center of Shanghai Institutes for Biological Sciences, Chinese Academy of Sciences, Shanghai, China) were used in cell transfection. Transient transfection was carried out using Lipofectamine 2000 (Life Technologies). Twenty-four hours after transfection, total RNAs were extracted using TRIzol Reagent (Life Technologies). After DNase I treatment (Thermo), the first strand cDNA was synthesized using RevertAid H Minus First Strand cDNA Synthesis Kit (Thermo). The wild-type and alternative splice variants were amplified with the following primers, P1 5′-CCTGGACAGCTTCCCCAA-3′ and P2 5′- CATGGCCACAGCTCTCTG-3′ (Figure 2A).

### RT-PCR Analysis of *AIR*E mRNA in Lymph Node

Total RNA was isolated from the lymph node needle biopsy of a heterozygous carrier (III-1; G/A) and a healthy control (IV-4; G/G) using ZR RNA MicroPrep Kit (Zymo Research). After DNase I treatment (Thermo), the first strand cDNA was synthesized using RevertAid H Minus First Strand cDNA Synthesis Kit (Thermo). The subsequent cDNA fragments were amplified with the same primers of minigene analysis. PCR products were detected by 2% agarose gel electrophoresis and confirmed by sequencing with the same primers.

The fluorescence density of the ethidium bromide bands obtained following gel electrophoresis of RT-CR products was quantified under unsaturated condition using ImageJ (National Institutes of Health) [29].

### *In vitro* Translation Assay

The construct expressing N-terminally tagged FLAG-AIRE was kindly provided by Dr. M. Matsumoto (University of Tokushima, Kuramoto, Tokushima, Japan) [30]. The Intron 3-retaining *AIRE* was generated by multiple ligating PCR fragments (Exon 1–3; Intron 3; Exon 4–14) into pCMV-Tag 2B (Agilent Technologies) digested with *Eco*R I and *Sal* I. The constructs were verified by DNA sequencing. The PCR fragments were amplified using primers:

Exon 1–3: AIRE-F1F CTGCAGGAATTCATGGCGACGGACG and AIRE-F1R TTGGGCTGGCGGTGCCCCTTG;

Intron 3: AIRE-F2F GTACCCTCCCTGCAGGGGAAGCCAG and AIRE-F2R CTGAATGGGGGAGCTGGGGGC;

Exon 4–14: AIRE-F3F GCTCTCAACTGAAGGCCAAGCCCCC and AIRE-F3R CTCGAGGTCGACTCAGGAGGGGAAGG.

For *in vitro* translation, wild-type *AIRE* construct and the intron 3-retaining *AIRE* construct were transfected into HEK293T respectively as described above. Antibodies used for immunoblotting: anti-FLAG (M20008S Abmart), anti-β-Actin (#4967 Cell Signaling Technologies).
